# Author Correction: Evolution of microscopic heterogeneity and dynamics in choline chloride-based deep eutectic solvents

**DOI:** 10.1038/s41467-022-28671-4

**Published:** 2022-02-22

**Authors:** Stephanie Spittle, Derrick Poe, Brian Doherty, Charles Kolodziej, Luke Heroux, Md Ashraful Haque, Henry Squire, Tyler Cosby, Yong Zhang, Carla Fraenza, Sahana Bhattacharyya, Madhusudan Tyagi, Jing Peng, Ramez A. Elgammal, Thomas Zawodzinski, Mark Tuckerman, Steve Greenbaum, Burcu Gurkan, Clemens Burda, Mark Dadmun, Edward J. Maginn, Joshua Sangoro

**Affiliations:** 1 Department of Chemical and Biomolecular Engineering, University of Tennessee, Knoxville, TN 37996 USA; 2 Department of Chemical and Biomolecular Engineering, University of Notre Dame, Notre Dame, IN 46556 USA; 3 Department of Chemistry, New York University, New York, NY 10003 USA; 4 Department of Chemistry, Case Western Reserve University, Cleveland, OH 44106 USA; 5 Department of Materials Science and Engineering, University of Tennessee, Knoxville, TN 37996 USA; 6 Oak Ridge National Laboratory, Neutron Sciences Division, Oak Ridge, TN 37830 USA; 7 Department of Chemistry, University of Tennessee, Knoxville, TN 37996 USA; 8 Department of Chemical and Biomolecular Engineering, Case Western Reserve University, Cleveland, OH 44106 USA; 9 School of Mathematics and Sciences, University of Tennessee Southern, Pulaski, TN 44106 USA; 10 Department of Physics and Astronomy, Hunter College, New York, NY 10065 USA; 11 NIST Center for Neutron Research, Gaithersburg, MD 20899 USA; 12 Department of Materials Science and Engineering, University of Maryland, College Park, MD 20742 USA; 13 School of Materials Science and Engineering, Beihang University, Beijing, China; 14 Courant Institute of Mathematical Science, New York University, New York, NY 10012 USA; 15 NYU-ECNU Center for Computational Chemistry at NYU Shanghai, Shanghai, China; 16 Oak Ridge National Laboratory, Chemical Sciences Division, Oak Ridge, TN 37830 USA

**Keywords:** Fluids, Structure of solids and liquids, Chemical physics

Correction to: *Nature Communications* 10.1038/s41467-021-27842-z, published online 11 January 2022.

The original version of this Article, and the original version of the Supplementary Information associated with this Article, omitted from the author list the 19^th^ author Clemens Burda, who is from the Department of Chemistry, Case Western Reserve University, Cleveland, OH, 44106, USA. The text ‘C.K. and C.B. provided the femtosecond transient-absorption spectroscopy experiments and analysis’ was already present in the Author Contributions. The author list has been corrected in both the PDF and HTML versions of the Article, and the HTML has been updated to include a corrected version of the Supplementary Information.

## Supplementary information

Updated Supplementary Information

