# Supplementary material for: Evolution of microscopic heterogeneity and dynamics in choline chloride-based deep eutectic solvents
Source: Nat Commun. 2022 Jan 11;13:219. doi: 10.1038/s41467-021-27842-z (PMC8752670; doi:10.1038/s41467-021-27842-z)
Supplement: Supplementary file 1 — Supplementary Information [file 41467_2021_27842_MOESM1_ESM.pdf]

Evolution of Microscopic Heterogeneity and Dynamics in Choline Chloride-based Deep Eutectic Solvents

Stephanie Spittle[1,+], Derrick Poe[2,+], Brian Doherty[3], Charles Kolodziej[4], Luke Heroux[5,6], Md Ashraful Haque[7], Henry Squire[8], Tyler Cosby[9], Yong Zhang[2], Carla Fraenza[10], Sahana Bhattacharyya[10], Madhu Tyagi[11], Jing Peng[12], Ramez A. Elgammal[1], Thomas Zawodzinski[1], Mark Tuckerman[3,13,14], Steve Greenbaum[10], Burcu Gurkan[8], Clemens Burda[4], Mark Dadmun[7,15], Edward J. Maginn[2,*], and Joshua Sangoro[1,*]

[1]Department of Chemical and Biomolecular Engineering, University of Tennessee, Knoxville, TN 37996, USA
[2]Department of Chemical and Biomolecular Engineering, University of Notre Dame, Notre Dame, IN 46556, USA
[3]Department of Chemistry, New York University, New York, NY 10003
[4]Department of Chemistry, Case Western Reserve University, Cleveland, OH 44106, USA
[5]Department of Materials Science and Engineering, University of Tennessee, Knoxville, TN 37996, USA
[6]Oak Ridge National Laboratory, Neutron Sciences Division, Oak Ridge, TN 37830, USA
[7]Department of Chemistry, University of Tennessee, Knoxville, TN 37996, USA
[8]Department of Chemical and Biomolecular Engineering, Case Western Reserve University, Cleveland, OH 44106, USA
[9]School of Mathematics and Sciences, University of Tennessee Southern, Pulaski, TN 44106, USA
[10]Department of Physics and Astronomy, Hunter College, New York, NY 10065, USA
[11]National Institute of Standards and Technology, Gaithersburg, MD 20899, USA
[12]School of Materials Science and Engineering, Beihang University, Beijing, China
[13]Courant Institute of Mathematical Science, New York University, New York, NY 10012, USA
[14]NYU-ECNU Center for Computational Chemistry at NYU Shanghai, Shanghai, China
[15]Oak Ridge National Laboratory, Chemical Sciences Division, Oak Ridge, TN 37830, USA
[*]ed@nd.edu; jsangoro@utk.edu
[+]these authors contributed equally to this work

Table 1. Peak widths in Hz at 333 K as a function of composition.

	a	b	c	d	e	f	g	h	i
Glycerol	15	22	24	17	14				
5 mol% ChCl	30	26	13	22	24	9	14	18	11
10 mol% ChCl	19	21	7	14	21	7	9	11	13
20 mol% ChCl	15	17	5	13	14	9	8	13	14
33 mol% ChCl	14	15	6	11	12	10	8	12	17

Table 2. [1]H chemical shifts in ppm at 333 K as a function of composition.

	a	b	c	d	e	f	g	h	i
Glycerol	4.63	3.31	3.39	3.49	4.75				
5 mol% ChCl	4.65	3.37	3.47	3.56	4.76	3.03	3.44	3.86	4.97
10 mol% ChCl	4.62	3.39	3.49	3.58	4.73	3.07	3.47	3.88	4.96
20 mol% ChCl	4.54	3.39	3.48	3.57	4.64	3.09	3.45	3.88	4.93
33 mol% ChCl	4.46	3.34	3.42	3.52	4.51	3.11	3.39	3.84	4.91

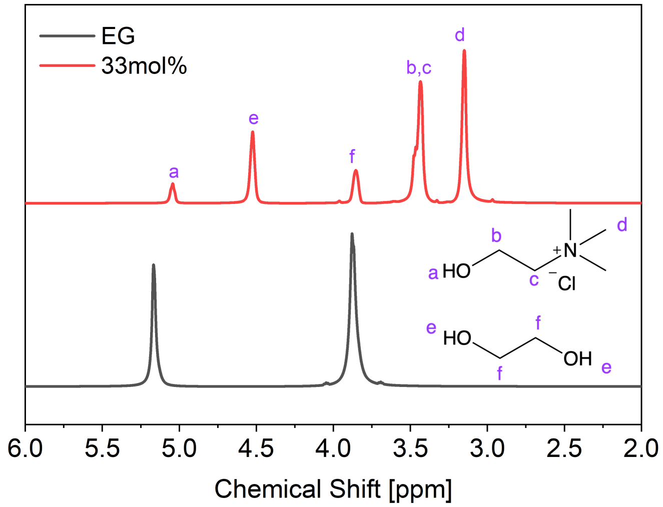

Figure 1. ^{1}H NMR spectra for 0 and 33mol% ChCl in ethylene glycol at 333 K.

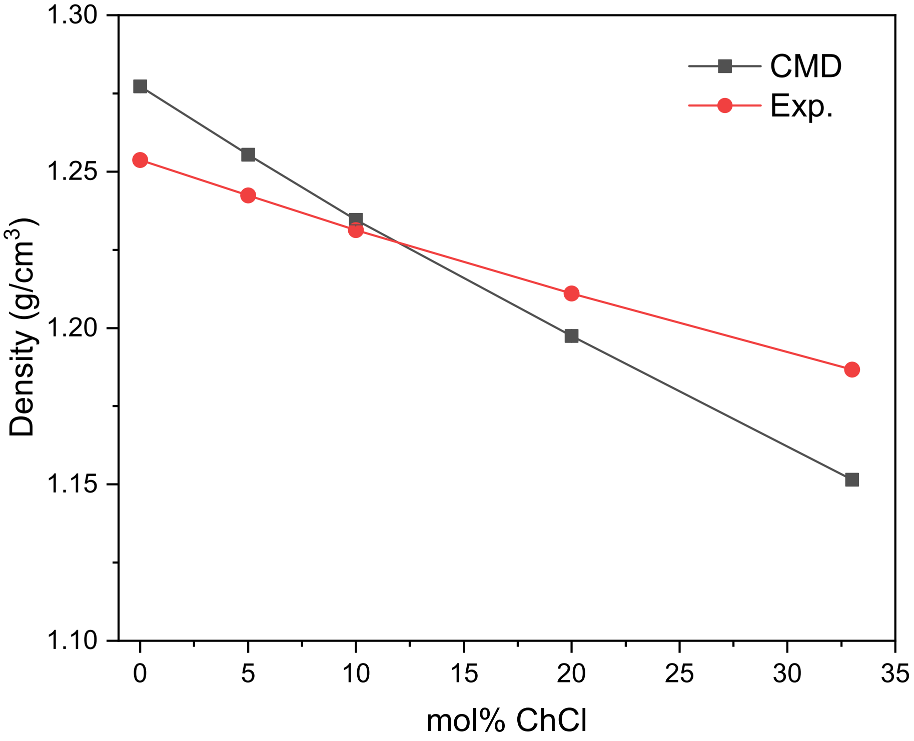

Figure 2. Density as a function of mol% Choline Chloride in Glycerol at 300K for CMD and experimental methods.

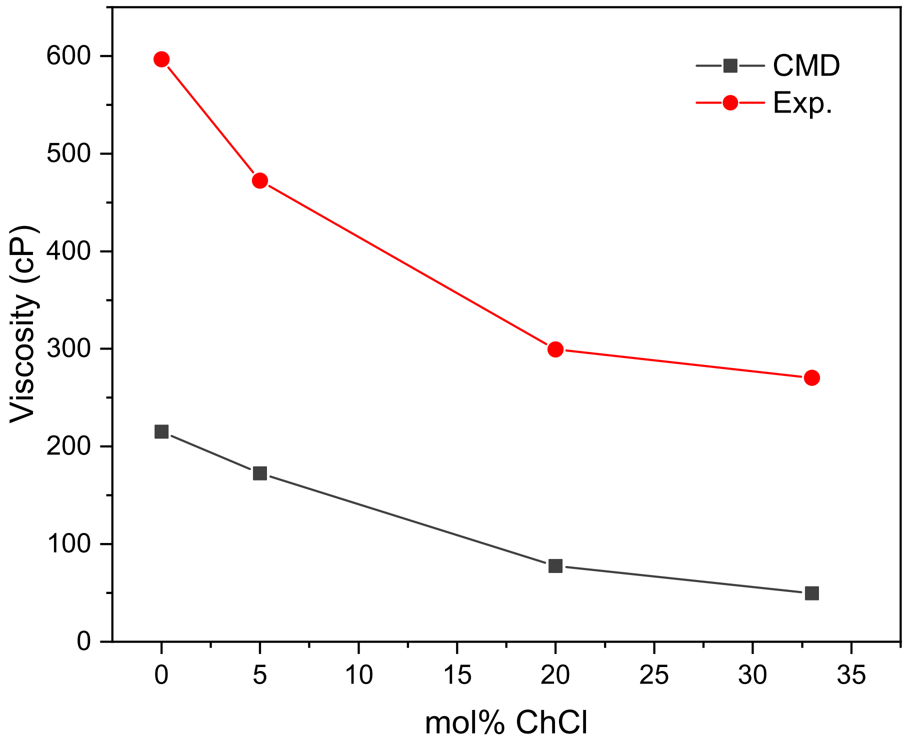

Figure 3. Viscosity as a function of mol% Choline Chloride in Glycerol at 300K for CMD and experimental methods.

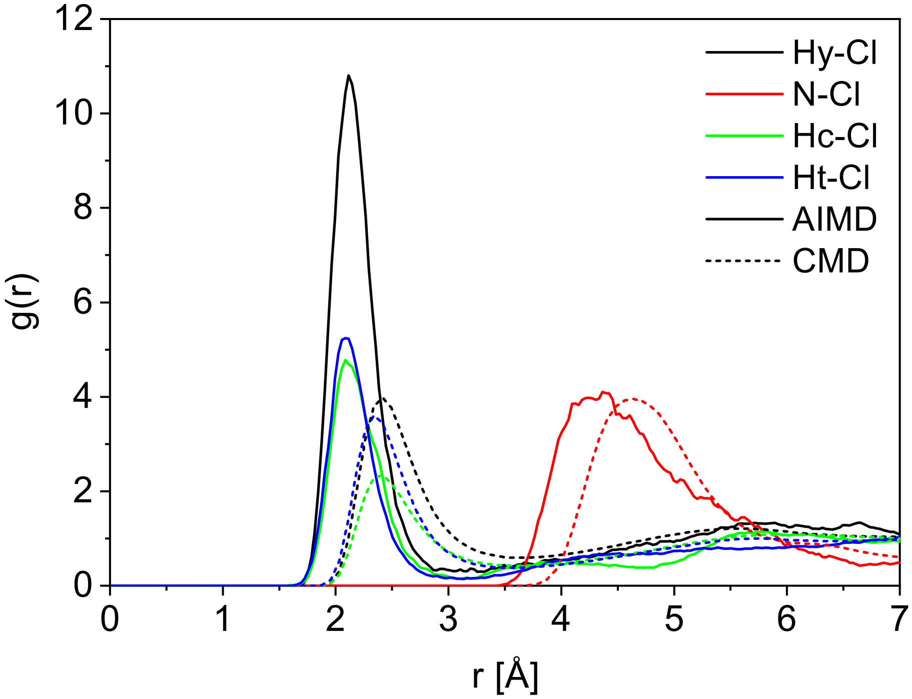

Figure 4. Partial RDFs for site–site interactions with chloride from CMD and AIMD simulations at 400K.

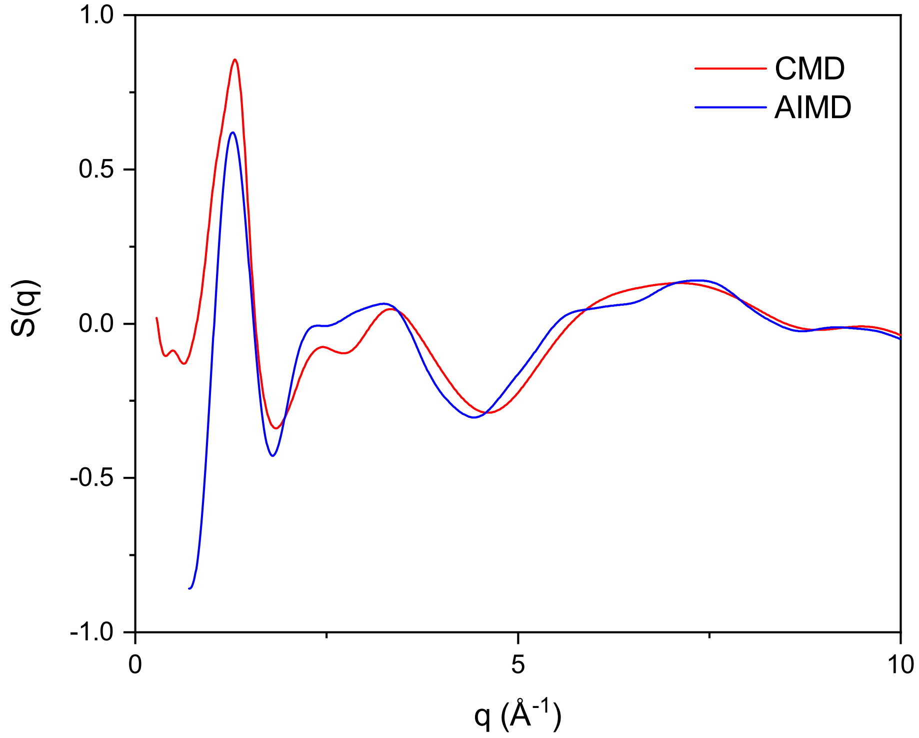

Figure 5. Comparison of CMD and AIMD simulated neutron scattering $S(q)$ versus q for 33 mol% ChCl in Glycerol at 400K.

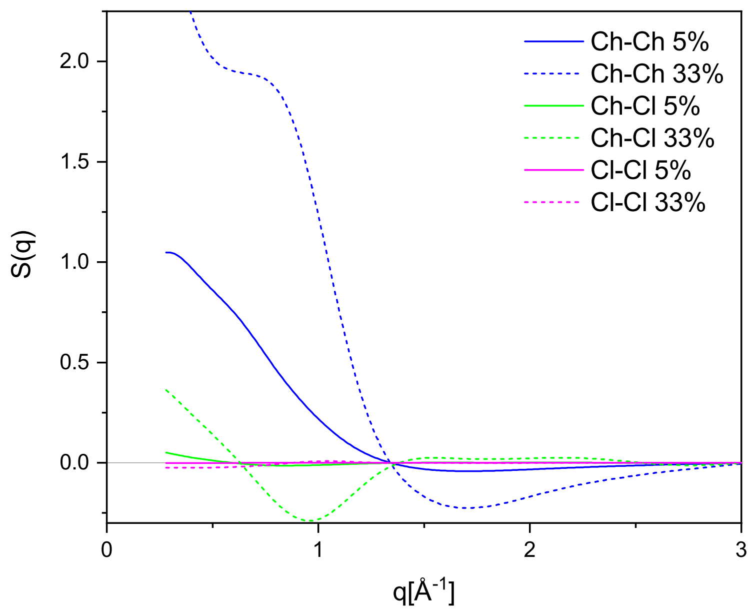

Figure 6. The structure factors associated with the ionic species obtained from neutron scattering for fully deuterated ChCl/glycerol mixtures.

Table 3. Full coordination numbers and standard deviation of coordination numbers derived from CMD simulations at 300K for 0, 5, 10, 20, and 33 mol% ChCl in Glycerol.

		0 mol%		5 mol%		10 mol%		20 mol%		33 mol%	
	site-site	N_{coor}	stdev	N_{coor}	stdev	N_{coor}	stdev	N_{coor}	stdev	N_{coor}	stdev
Gly-Gly	Oc-Hc	0.30	0.0005	0.28	0.002	0.26	0.0013	0.23	0.0027	0.31	0.0013
	Oc-Ht	0.53	0.0016	0.50	0.002	0.46	0.0032	0.39	0.0022	0.08	0.0009
	Ot-Hc	0.32	0.0008	0.31	0.001	0.29	0.0012	0.25	0.0011	0.42	0.0023
	Ot-Ht	0.71	0.0008	0.65	0.001	0.61	0.0016	0.52	0.0023	0.10	0.0009
Cl-Gly	Cl-Hc	-	-	0.81	0.030	0.76	0.0103	0.67	0.0056	0.55	0.0086
	Cl-Ht	-	-	2.33	0.030	2.13	0.0199	1.82	0.0092	1.42	0.0111
Cl-Ch	Cl-Hy	-	-	0.11	0.011	0.21	0.0109	0.34	0.0059	0.50	0.0087
Gly-Ch	Oc-Hy	-	-	0.01	0.001	0.03	0.0011	0.05	0.0006	0.08	0.0009
	Ot-Hy	-	-	0.02	0.000	0.03	0.0004	0.06	0.0005	0.10	0.0009
Ch-Gly	Oy-Hc	-	-	0.09	0.008	0.09	0.0052	0.07	0.0051	0.05	0.0030
	Oy-Ht	-	-	0.11	0.014	0.10	0.0067	0.08	0.0007	0.05	0.0009
Ch-Ch	Oy-Hy	-	-	0.00	0.001	0.00	0.0008	0.01	0.0008	0.01	0.0002

Table 4. Coordination numbers and standard deviation of coordination numbers derived from three 6ns long CMD simulations at 340K for 33 mol% ChCl in Glycerol.

		33% 340K	
	site-site	Ncoor	stdev
Gly-Gly	Oc-Hc	0.17	0.0007
	Oc-Ht	0.28	0.0018
	Ot-Hc	0.20	0.0006
	Ot-Ht	0.38	0.0011
Cl-Gly	Cl-Hc	0.59	0.0026
	Cl-Ht	1.47	0.0075
Cl-Ch	Cl-Hy	0.51	0.0054
Gly-Ch	Oc-Hy	0.08	0.0002
	Ot-Hy	0.10	0.0006
Ch-Gly	Oy-Hc	0.04	0.0142
	Oy-Ht	0.05	0.0049
Ch-Ch	Oy-Hy	0.01	0.0037

The real and imaginary parts of complex permittivity and complex conductivity are shown in Figures 7-11 for 0, 5, 10, 20, and 33mol% ChCl in glycerol for one representative temperature. The fitting parameters for all temperatures are listed in Supplementary Tables 5-9. The derivative representation of the imaginary part of complex permittivity, described in the BDS methods, is also shown in the same plot as the imaginary part of complex permittivity. It is clear from this analysis that more information is obtained by using the derivative representation.

Figure 5a in the main text shows the structural relaxation rates from fs-TA, BDS, and DMS as a function of temperature with Vogel-Fulcher-Tammann (VFT) fits. The VFT equation is

$$\tau = \tau_0 exp(A/(T - T_0)) \qquad (1)$$

where τ_0, A, and T_0 are fitting parameters.

The data for the real part of complex permittivity of 33mol% ChCl in glycerol at 221 K from Faraone *et al.* is plotted in the top of Supplementary Figure 13. Data from this paper at 33mol% ChCl at 220 K is plotted as well for reference. In the bottom, the derivative analysis was formed to show that if considered, a secondary, slower process (the sub-α relaxation) in present. The electrode polarization occurs at lower frequencies in our data, which could be due to a larger sample thickness used. This unveils even more information for the secondary relaxation.

The fs-TA data for 33mol% ChCl in glycerol are shown in Supplementary Figure 14.

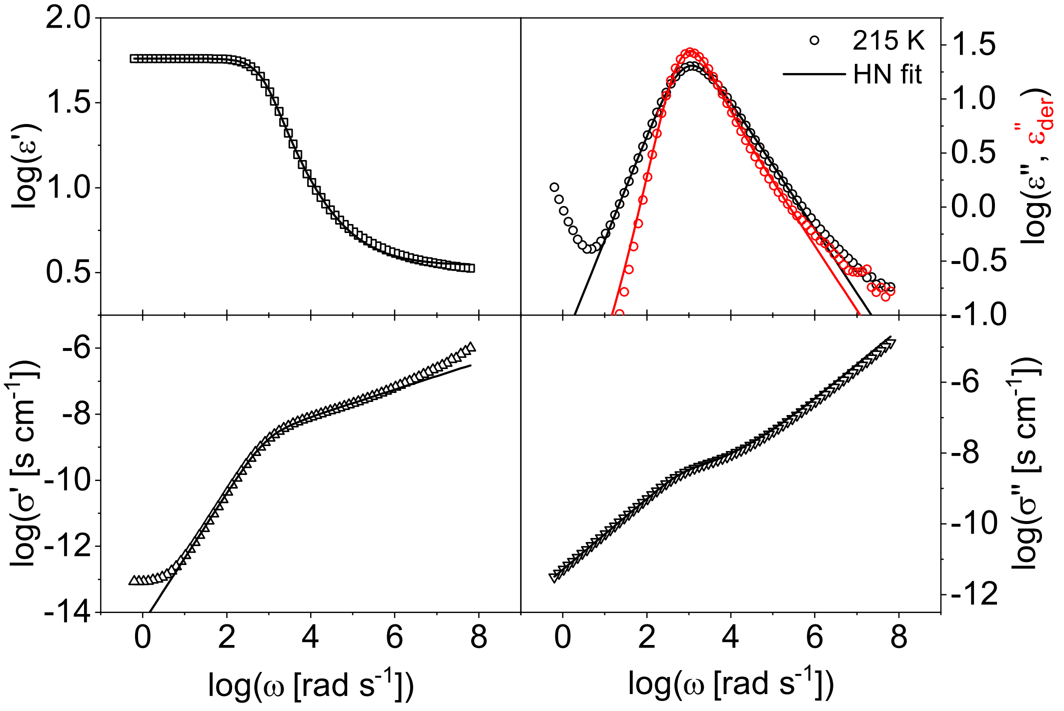

Figure 7. Real and imaginary parts of the complex dielectric function and complex conductivity function for glycerol at 215 K. The derivative representation of the dielectric loss is plotted in red. Solid lines are fits, which is a single Havriliak-Negami function.

Table 5. Fitting parameters for dielectric data of glycerol using a Havriliak-Negami function.

Temp. [K]	ε_∞	$\Delta\varepsilon$	τ_α [s]	β	γ
200	3.99	61.3	3.88E-01	0.90	0.67
205	3.89	57.7	4.19E-02	0.94	0.63
210	3.39	56.1	6.84E-03	0.96	0.61
215	3.50	54.2	1.28E-03	0.97	0.61
220	3.60	52.4	2.84E-04	0.97	0.62
225	3.60	50.7	7.44E-05	0.97	0.63
230	3.60	49.2	2.19E-05	0.97	0.64
235	3.70	47.5	7.19E-06	0.97	0.65
240	3.70	46.1	2.62E-06	0.97	0.66
245	3.70	44.6	1.05E-06	0.98	0.66
250	3.70	43.3	4.55E-07	0.98	0.66
255	3.69	41.9	2.04E-07	0.98	0.67
260	3.20	41.2	1.03E-07	0.98	0.66
265	3.30	39.8	5.15E-08	0.98	0.67
270	3.20	38.8	2.81E-08	0.98	0.68

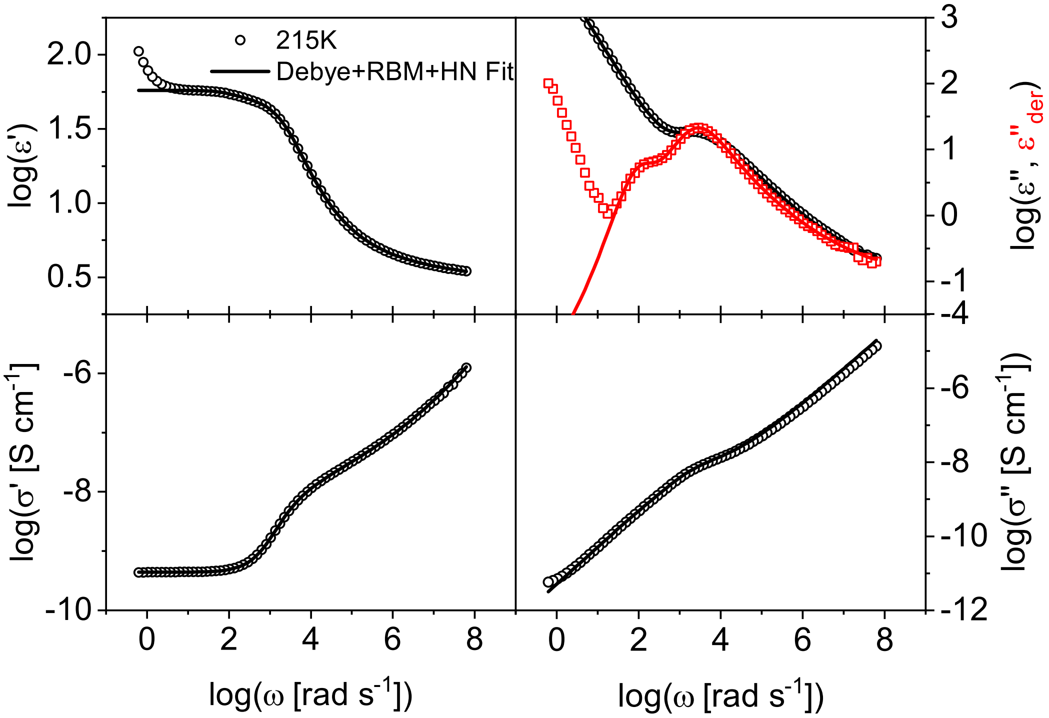

Figure 8. Real and imaginary parts of the complex dielectric function and complex conductivity function for 5mol% ChCl in glycerol at 215 K. The derivative representation of the dielectric loss is plotted in red. Solid lines are fits, which is are a combination of a Debye function, Havriliak-Negami , and a Random Barrier Model.

Table 6. Fitting parameters for dielectric data of 5mol% ChCl in glycerol using a Debye, Havriliak-Negami function, and Random Barrier Model.

Temp. [K]	ε_∞	$\Delta\varepsilon_{slow}$	τ_{slow} [s]	$\Delta\varepsilon_\alpha$	τ_α [s]	β	γ	σ_0 [S cm^{-1}]	τ_{ion} [s]
205	1.96	6.31	1.92E-01	43.8	1.09E-02	0.84	0.79	2.11E-11	8.20E-02
210	2.00	6.12	3.66E-02	42.1	1.94E-03	0.85	0.77	1.06E-10	1.56E-02
215	1.92	5.82	8.53E-03	40.3	4.18E-04	0.87	0.77	4.42E-10	3.82E-03
220	2.00	5.63	2.18E-03	39.1	1.08E-04	0.88	0.76	1.61E-09	9.88E-04
225	2.05	5.44	6.21E-04	37.8	3.17E-05	0.90	0.75	5.25E-09	2.86E-04
230	1.95	5.09	2.11E-04	36.3	1.00E-05	0.90	0.75	1.58E-08	9.84E-05
235	2.30	5.10	6.59E-05	35.9	3.60E-06	0.91	0.75	4.38E-08	2.82E-05
240	1.93	4.71	2.65E-05	33.7	1.39E-06	0.93	0.74	1.11E-07	1.35E-05
245	2.00	4.71	1.09E-05	32.8	6.03E-07	0.94	0.72	2.62E-07	5.22E-06
250	2.00	4.62	4.59E-06	33.2	2.84E-07	0.94	0.70	5.84E-07	1.82E-06

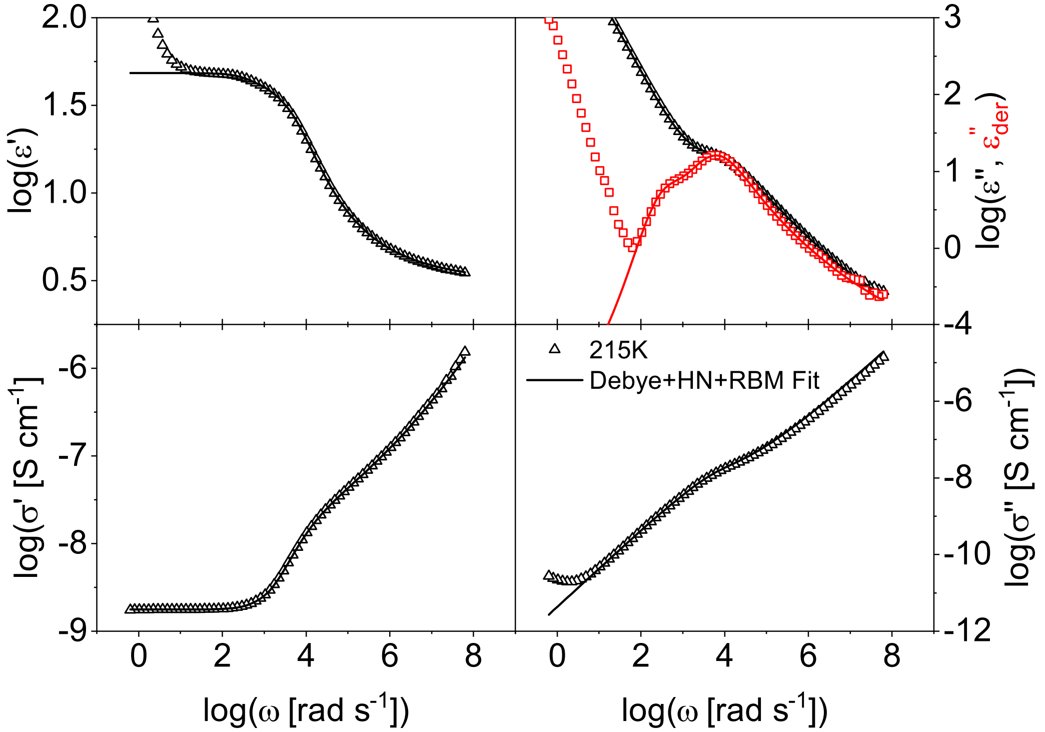

Figure 9. Real and imaginary parts of the complex dielectric function and complex conductivity function for 10mol% ChCl in glycerol at 215 K. The derivative representation of the dielectric loss is plotted in red. Solid lines are fits, which is are a combination of a Debye function, Havriliak-Negami , and a Random Barrier Model.

Table 7. Fitting parameters for dielectric data of 10mol% ChCl in glycerol using a Debye, Havriliak-Negami function, and Random Barrier Model.

Temp. [K]	ε_∞	$\Delta\varepsilon_{slow}$	τ_{slow} [s]	$\Delta\varepsilon_\alpha$	τ_α [s]	β	γ	σ_0 [S cm^{-1}]	τ_{ion} [s]
200	2.13	5.88	2.44E-01	37.7	2.64E-02	0.75	0.87	1.85E-11	7.81E-02
205	2.00	5.45	4.55E-02	35.9	4.02E-03	0.75	0.89	9.93E-11	1.55E-02
210	2.00	5.54	9.49E-03	34.2	7.96E-04	0.78	0.86	4.53E-10	3.30E-03
215	2.00	5.58	2.26E-03	32.6	1.88E-04	0.80	0.84	1.76E-09	8.27E-04
220	2.00	5.46	6.36E-04	31.2	5.23E-05	0.82	0.83	6.00E-09	2.37E-04
225	2.00	5.03	2.04E-04	30.3	1.60E-05	0.82	0.84	1.86E-08	7.52E-05
230	2.00	4.99	6.56E-05	28.9	5.45E-06	0.84	0.82	5.30E-08	2.58E-05
235	2.00	4.76	2.58E-05	27.8	2.11E-06	0.84	0.83	1.33E-07	1.03E-05
240	2.00	4.68	9.55E-06	26.7	8.23E-07	0.87	0.80	3.44E-07	3.80E-06
245	2.94	4.09	4.24E-06	29.5	4.07E-07	0.86	0.77	7.85E-07	6.77E-07
250	2.00	3.78	1.90E-06	26.8	1.77E-07	0.85	0.80	1.67E-06	6.09E-07

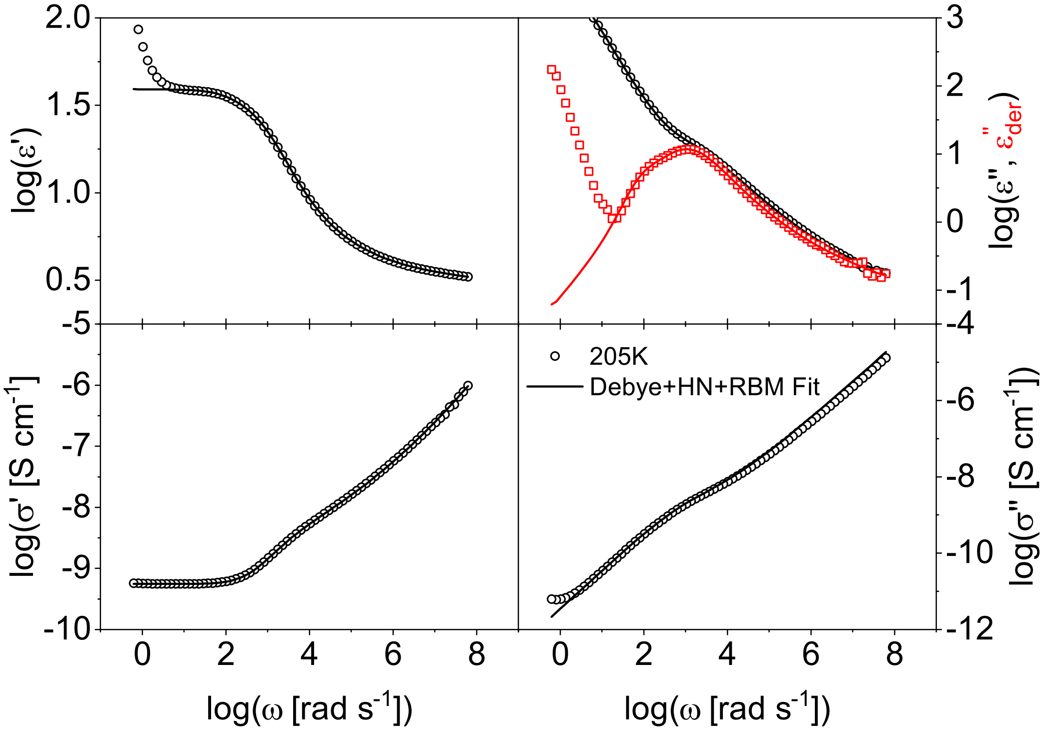

Figure 10. Real and imaginary parts of the complex dielectric function and complex conductivity function for 20mol% ChCl in glycerol at 205 K. The derivative representation of the dielectric loss is plotted in red. Solid lines are fits, which is are a combination of a Debye function, Havriliak-Negami , and a Random Barrier Model.

Table 8. Fitting parameters for dielectric data of 20mol% ChCl in glycerol using a Debye, Havriliak-Negami function, and Random Barrier Model.

Temp. [K]	ε_∞	$\Delta\varepsilon_{slow}$	τ_{slow} [s]	$\Delta\varepsilon_\alpha$	τ_α [s]	β	γ	σ_0 [S cm^{-1}]	τ_{ion} [s]
200	2.13	5.88	2.44E-01	37.7	2.64E-02	0.75	0.87	1.85E-11	7.81E-02
205	2.00	5.45	4.55E-02	35.9	4.02E-03	0.75	0.89	9.93E-11	1.55E-02
210	2.00	5.54	9.49E-03	34.2	7.96E-04	0.78	0.86	4.53E-10	3.30E-03
215	2.00	5.58	2.26E-03	32.6	1.88E-04	0.80	0.84	1.76E-09	8.27E-04
220	2.00	5.46	6.36E-04	31.2	5.23E-05	0.82	0.83	6.00E-09	2.37E-04
225	2.00	5.03	2.04E-04	30.3	1.60E-05	0.82	0.84	1.86E-08	7.52E-05
230	2.00	4.99	6.56E-05	28.9	5.45E-06	0.84	0.82	5.30E-08	2.58E-05
235	2.00	4.76	2.58E-05	27.8	2.11E-06	0.84	0.83	1.33E-07	1.03E-05
240	2.00	4.68	9.55E-06	26.7	8.23E-07	0.87	0.80	3.44E-07	3.80E-06
245	2.94	4.09	4.24E-06	29.5	4.07E-07	0.86	0.77	7.85E-07	6.77E-07
250	2.00	3.78	1.90E-06	26.8	1.77E-07	0.85	0.80	1.67E-06	6.09E-07

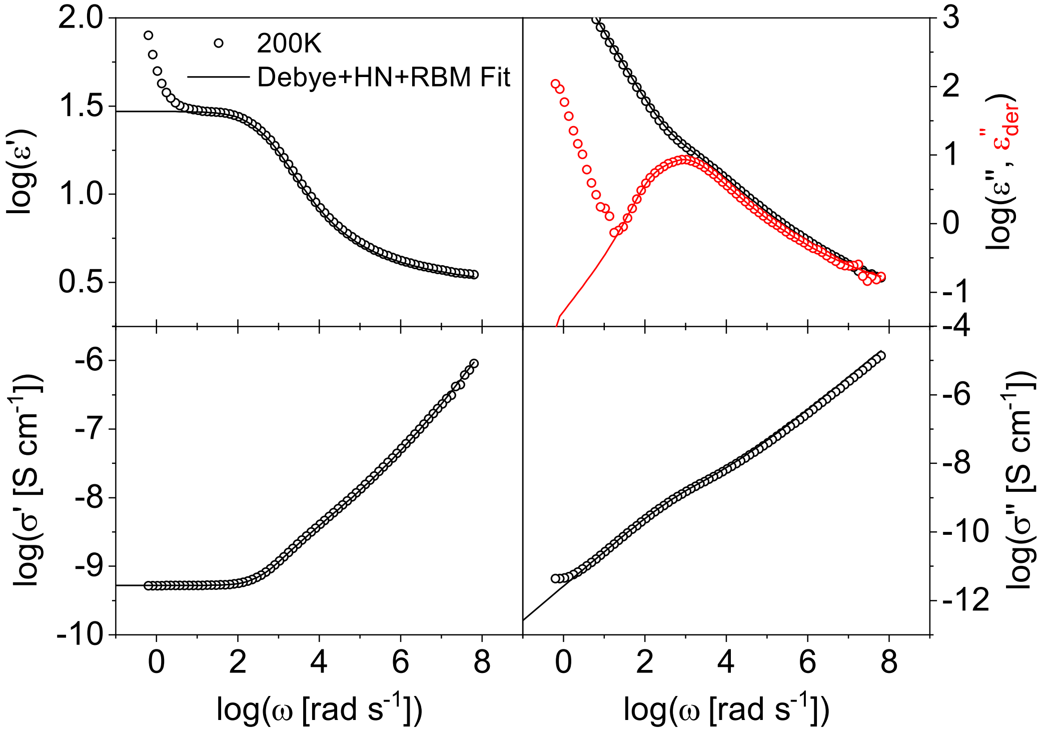

Figure 11. Real and imaginary parts of the complex dielectric function and complex conductivity function for 33mol% ChCl in glycerol at 200 K. The derivative representation of the dielectric loss is plotted in red. Solid lines are fits, which is are a combination of a Debye function, Havriliak-Negami , and a Random Barrier Model.

Table 9. Fitting parameters for dielectric data of 33mol% ChCl in glycerol using a Debye, Havriliak-Negami function, and Random Barrier Model.

Temp. [K]	ε_∞	$\Delta\varepsilon_{slow}$	τ_{slow} [s]	$\Delta\varepsilon_\alpha$	τ_α [s]	β	γ	σ_0 [S cm^{-1}]	τ_{ion} [s]
190	2.33	2.57	1.85E-01	19.0	5.82E-02	0.74	0.76	1.72E-11	7.73E-02
195	2.33	2.81	2.76E-02	19.1	8.00E-03	0.76	0.73	1.23E-10	1.05E-02
200	2.25	2.31	5.68E-03	18.0	1.43E-03	0.74	0.79	5.58E-10	2.33E-03
205	2.26	2.49	1.27E-03	17.9	3.02E-04	0.76	0.76	2.37E-09	5.41E-04
210	2.25	2.08	3.65E-04	17.0	7.95E-05	0.76	0.79	7.99E-09	1.54E-04
215	2.45	2.39	1.04E-04	17.4	2.47E-05	0.80	0.72	2.77E-08	3.75E-05
220	2.25	2.02	3.64E-05	15.7	7.85E-06	0.78	0.79	7.38E-08	1.58E-05
225	2.24	2.32	1.29E-05	15.3	2.81E-06	0.81	0.76	2.10E-07	5.60E-06
230	2.25	1.14	5.85E-06	15.8	1.28E-06	0.78	0.79	4.90E-07	2.18E-06
235	2.24	1.67	2.24E-06	15.3	5.68E-07	0.84	0.70	1.22E-06	8.39E-07
300	2.24	1.60	2.87E-09	14.5	6.59E-10	0.79	0.78	7.99E-04	1.84E-09

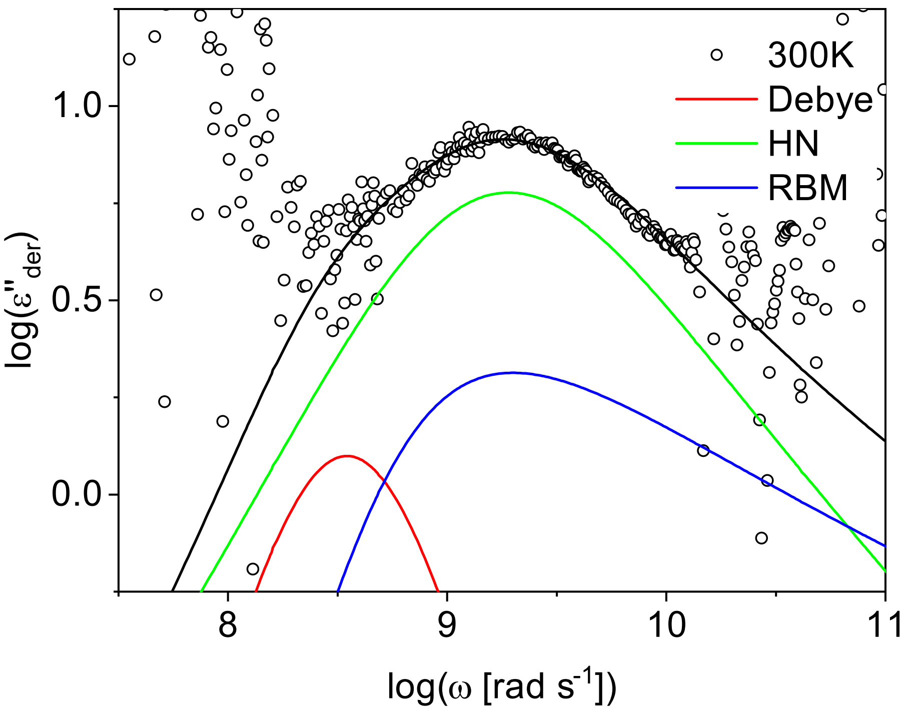

Figure 12. The derivative representation of the dielectric loss for 33mol% ChCl in glycerol at room temperature plotted as a function of radial frequency.

	τ_0 [s]	T_0 [K]	A
glycerol	1.33E-15	127	2384
5mol% ChCl	1.93E-16	117	2755
10mol% ChCl	1.17E-16	114	2821
20mol% ChCl	5.04E-16	113	2592
33mol% ChCl	5.27E-16	109	2564

Table 10. Vogel-Fulcher-Tammann fit parameters for the structural relaxation rates for 0, 5, 10, 20, and 33mol% ChCl in glycerol.

39 The parameters for the VFT fits from Figure 5a are in Supplementary Table 10.

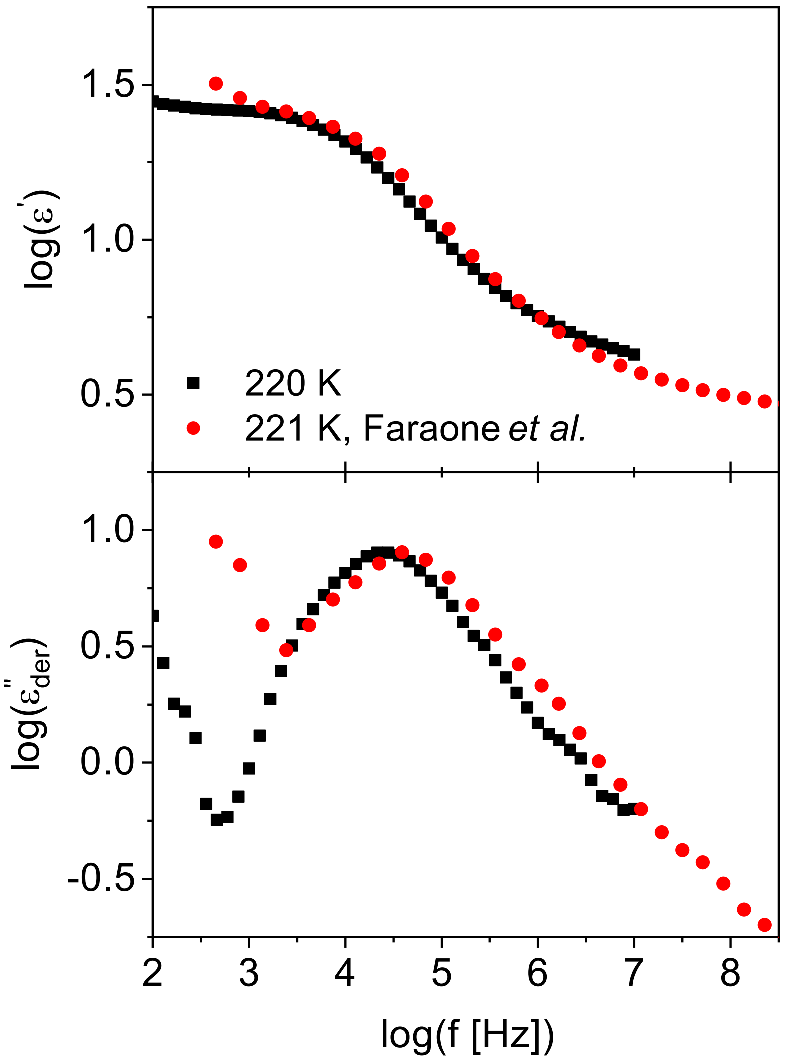

Figure 13. Top: Real part of complex permittivity for 33mol% ChCl in glycerol obtained for this paper and from Faraone *et al.* and 220 and 221 K respectively. Bottom: The derivative representation of the dielectric loss at 220 and 221 K from this paper and Faraone *et al.* respectively.

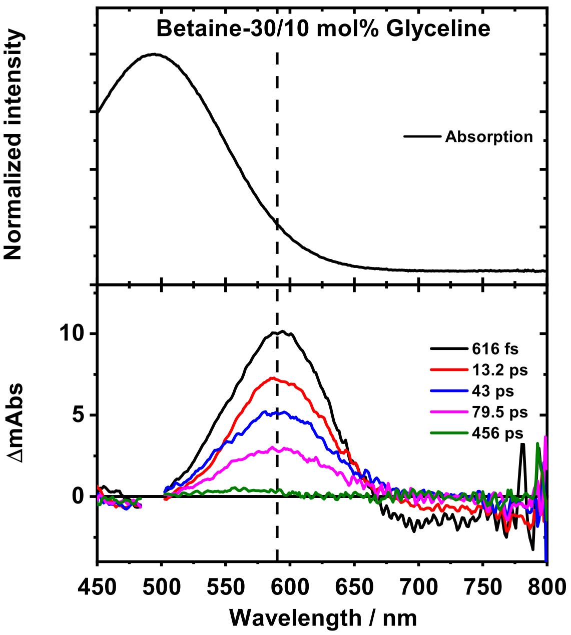

(a)

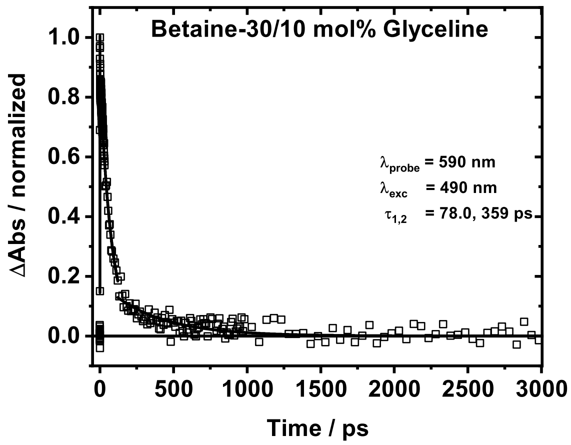

(b)

Figure 14. (a) Top panel: Steady-state UV-visible absorption of betaine-30, bottom panel: femtosecond transient absorption spectra at indicated delay times. (b) fs-TA kinetics of betaine-30 in glycerol at a probe wavelength of 566 nm. (c) Top panel: Steady-state UV-visible absorption of betaine-30, bottom panel: femtosecond transient absorption spectra at indicated delay times. (d) fs-TA kinetics of betaine-30 in 10 mol% Glyceline at a probe wavelength of 590 nm.

Data presented in Supplementary Table 12 is done with a force field tuned to simulate ethylene glycol and choline chloride mixtures and is undergoing further refinement to better capture fine, concentration dependent dynamics trends.

Table 11. Coefficients (time in ns) fitted to Equation 5 for the dipole moment rotational dynamics based on CMD simulations at 298 K.

	b_1	α_1	b_2	α_2	τ_1	τ_2	R^2
Choline							
0 mol%	-	-	-	-	-	-	-
5 mol%	0.4695	0.9690	0.5305	0.6212	0.4277 ±0.0993	0.0172 ±0.0099	0.9970
10 mol%	0.4308	0.9860	0.5692	0.6892	0.3739 ±0.0558	0.0184 ±0.0041	0.9984
20 mol%	0.4555	0.9659	0.5445	0.6846	0.2503 ±0.0228	0.0127 ±0.0017	0.9988
33 mol%	0.4607	0.9816	0.4258	0.7546	0.1681 ±0.0109	0.0092 ±0.0008	0.9977
Glycerol							
0 mol%	0.4748	0.9391	0.5252	0.6923	0.1403 ±0.0082	0.0016 ±0.0001	0.9963
5 mol%	0.4916	0.9142	0.5084	0.6394	0.1330 ±0.0081	0.0013 ±0.0006	0.9970
10 mol%	0.4881	0.9161	0.5119	0.6806	0.1286 ±0.0069	0.0014 ±0.0001	0.9971
20 mol%	0.4908	0.9150	0.5092	0.6919	0.1109 ±0.0061	0.0012 ±0.0005	0.9964
33 mol%	0.4599	0.9370	0.5143	0.7111	0.0924 ±0.0029	0.0014 ±0.0001	0.9959

Table 12. The average slow (τ_1) and fast (τ_2) characteristic time coefficients of dipole relaxation obtained from CMD simulations for choline and ethylene glycol at various ChCl concentrations in ethylene glycol.

		τ_1(ps)	τ_2(ps)
0% ChCl	EG	3.3	0.3
5% ChCl	Choline	25.0	2.2
	EG	4.9	0.3
10% ChCl	Choline	46.0	5.6
	EG	7.2	0.3
16.7% ChCl	Choline	84.1	9.3
	EG	12.2	0.4
20% ChCl	Choline	149.1	15.8
	EG	15.5	0.4
25% ChCl	Choline	210.1	17.8
	EG	22.6	0.5
33% ChCl	Choline	486.4	30.7
	EG	46.4	0.6

The Stokes-Einstein relation can be written as

$$D = \frac{k_b\, T}{6\pi\eta r} \tag{2}$$

where D is the self-diffusion coefficient, k_b is the Boltzmann constant, T is temperature, η is viscosity, and r is radius of the particle. Using Supplementary Equation 2, if we calculate $D\eta/T$, we obtain what should be $k_b/(6\pi r)$, which should be constant if the relation holds. As seen in Supplementary Figure 15, these mixtures do not abide by the Stokes-Einstein relation.

Table 13. Standard deviation of hydrogen bond dynamics time constants (ps) derived from CMD simulations at 300K for 0, 5, 10, 20, and 33 mol% ChCl in Glycerol.

		0 mol%		5 mol%		10 mol%		20 mol%		33 mol%	
	site-site	τ_f	τ_b	τ_f	τ_b	τ_f	τ_b	τ_f	τ_b	τ_f	τ_b
Gly-Gly	Oc-Hc	4.8	11.4	5.2	15.3	4.9	11.3	6.7	24.2	5.8	13.6
	Oc-Ht	4.8	11.0	2.6	10.5	7.4	15.9	4.0	12.7	4.9	8.9
	Ot-Hc	4.3	10.7	2.9	7.3	6.0	13.4	5.8	8.6	4.9	14.3
	Ot-Ht	4.8	11.4	3.7	6.3	6.8	9.5	7.2	13.7	4.8	8.3
Cl-Gly	Cl-Hc	-	-	12.5	12.2	7.8	7.6	5.6	7.0	5.1	6.1
	Cl-Ht	-	-	11.2	7.4	9.7	6.6	9.9	6.3	8.7	5.2
Cl-Ch	Cl-Hy	-	-	23.3	24.0	22.2	24.9	5.9	7.5	4.8	4.3
Gly-Ch	Oc-Hy	-	-	6.6	20.5	3.3	12.9	4.4	9.5	1.9	4.3
	Ot-Hy	-	-	1.1	2.0	4.3	12.5	5.8	9.1	3.4	5.8
Ch-Gly	Oy-Hc	-	-	8.5	63.1	5.2	23.3	4.1	48.2	3.1	32.0
	Oy-Ht	-	-	5.6	99.5	1.7	77.7	3.7	51.8	2.4	45.1
Ch-Ch	Oy-Hy	-	-	3.3	16.6	5.5	18.7	3.0	18.3	2.0	11.8

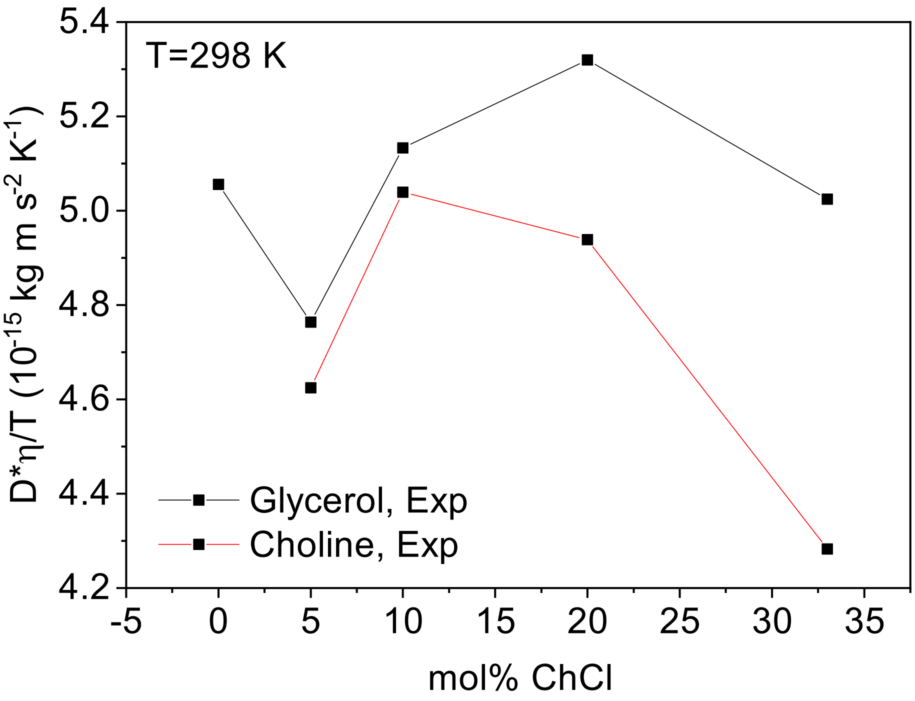

Figure 15. The self diffusion coefficient of choline and glycerol respectively multipled by viscosity divided by temperature at 298 K plotted versus mol% ChCl to test the Stokes-Einstein relation.

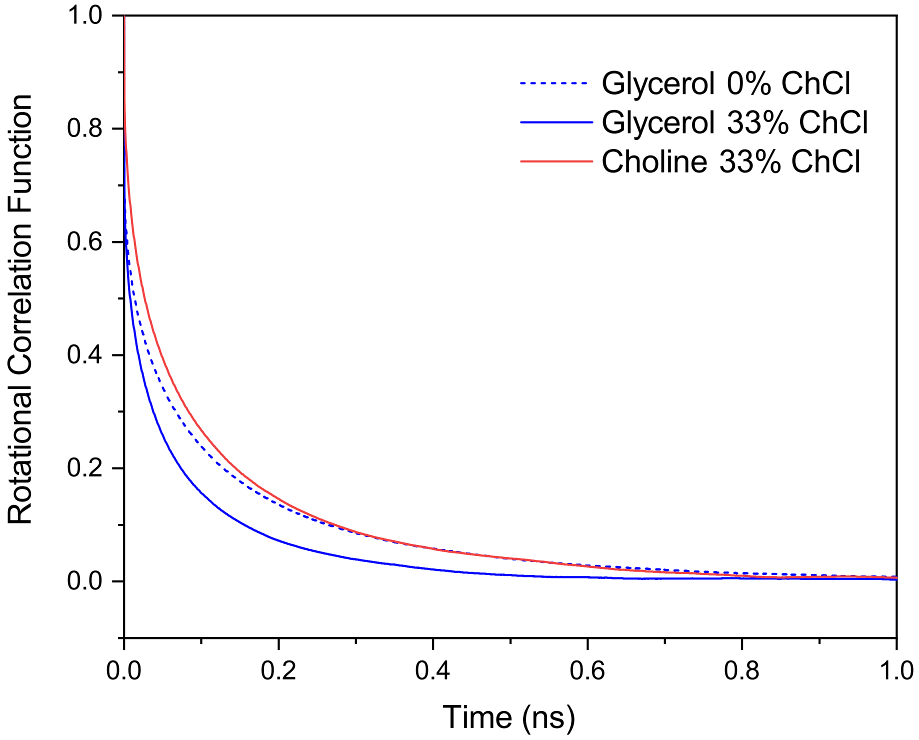

Figure 16. Normalized dipole moment rotational correlation function of glycerol and choline in Glyceline (solid) and pure glycerol (dashed) at 300K

The dielectric data of glycerol, only exhibiting the structural, α-relaxation can be described by a single Havriliak-Negami (HN) function, shown in Supplementary Equation 3:

$$\varepsilon^*(\omega) = \varepsilon_\infty + \frac{(\Delta\varepsilon)_\alpha}{(1+(i\omega\tau_\alpha)^\gamma)^\beta} \tag{3}$$

where ε_∞ is the high frequency plateau, $\Delta\varepsilon$ is the dielectric strength, τ is the mean relaxation time, σ_0 is the dc conductivity, and γ and β are shape parameters. The HN function is a modified Debye function. A Debye function describes a system of non-interacting systems, and the HN version has added shape parameters to account for broadening in the distribution of relaxation times. Above 5mol% ChCl, the mixtures can be well described by a combination of a Debye function for the slow process, a HN function for the α-relaxation, and a Random Barrier Model (RBM) for the ion dynamics. The RBM describes an ion hopping motion where the ions overcome a potential energy barrier to jump to the next spot. The full equation is:

$$\varepsilon^*(\omega) = \varepsilon_\infty + \frac{(\Delta\varepsilon)_{Debye}}{1+i\omega\tau_{Debye}} + \frac{\sigma_0\tau_{ion}}{\varepsilon_0(ln(1+i\omega\tau_{ion}))} + \frac{(\Delta\varepsilon)_\alpha}{(1+(i\omega\tau_\alpha)^\gamma)^\beta} \tag{4}$$

where σ_0 is the dc ionic conductivity and τ_{ion} is the ion hopping time. The subscripts Debye, ion, and α denote the relaxation the parameter is associated with.

The mechanical spectroscopy data were fit with two Cole-Davidson (CD) modified Maxwell models for the structural relaxation and the slow relaxation associated with hydrogen bonding structures as described in Supplementary Equation 5:

$$\eta^*(\omega) = \frac{G_{\infty,slow}}{\omega}\left[1 - \frac{1}{(1+i\omega\tau_{slow})^\gamma}\right] + \frac{G_{\infty,\alpha}}{\omega}\left[1 - \frac{1}{(1+i\omega\tau_\alpha)^\gamma}\right] \tag{5}$$

where G_∞ is the high frequency plateau in G', τ is the relaxation time, γ is the shape parameter, and the subscripts slow and α denote the distinct slow and α relaxations observed in the spectra. A Maxwell model describes a viscoelastic material, and the CD version adds the shape parameter exponent, γ, to account for broadening of the curves.
